# Personalized decision-making through AI solutions in pediatric emergency medicine: Focusing on febrile children

**DOI:** 10.1371/journal.pdig.0001080

**Published:** 2025-11-03

**Authors:** Lina Jankauskaite, Urte Oniunaite, Rimantas Kevalas

**Affiliations:** 1 Department of Pediatrics, Faculty of Medicine, Lithuanian University of Health Sciences, Kaunas, Lithuania; 2 Faculty of Medicine, Lithuanian University of Health Sciences, Institute of Physiology and Pharmacology, Kaunas, Lithuania; Durham University, UNITED KINGDOM OF GREAT BRITAIN AND NORTHERN IRELAND

## Abstract

Pediatric emergency medicine (PEM) presents unique challenges due to the diverse developmental stages and medical conditions of young patients. The increasing patient load and nonurgent referrals to pediatric emergency departments (PEDs) emphasize the need for personalized decision-making approaches. These approaches must accommodate the complexities of pediatric care while fostering collaboration between healthcare providers and families. Integrating artificial intelligence (AI) into healthcare settings can transform PEM by enhancing diagnostic accuracy, customizing treatments, and optimizing resource allocation. AI technologies leverage vast datasets, including electronic health records and genetic profiles, to generate personalized diagnostic and treatment plans. Machine learning algorithms can identify patterns in complex data, facilitating early disease detection and precise interventions. This literature review analyzes the role of AI in supporting pediatric emergency care through diagnostic assistance, predictive modeling for febrile disease progression, and outcome optimization. It also highlights the challenges of applying AI in PEM, including data limitations and the need for algorithmic transparency. By addressing these challenges, AI has the potential to revolutionize personalized care in pediatric emergency settings, ultimately improving patient outcomes and care delivery.

## Introduction

Pediatric emergency medicine (PEM) facilities are specific with regard to a variety of different conditions, procedures, and extremely severe cases. However, with increasing numbers of patients and a load of less urgent referrals to the pediatric emergency department (PED) [1], there is a necessity for different personalized decision-making solutions. This is crucial due to the diverse needs of young patients, which necessitate tailored approaches. Children present a wide range of developmental stages and medical conditions, requiring nuanced decision-making frameworks that consider individual circumstances [2]. Moreover, families desire active participation in medical decisions, emphasizing the need for healthcare providers to engage meaningfully rather than adopting a passive approach. Thus, healthcare providers and families should collaboratively navigate complex medical choices and issues during the stay and after a visit to PED. In addition, providers should contextualize medical information to help families make informed choices, particularly in high-stakes situations [3]. Nevertheless, as personalized decision-making could introduce challenges, such as the potential information overload for the families, the balance between autonomy and the need for expert guidance remains crucial in ensuring optimal outcomes in PEM.

Integrating artificial intelligence (AI) in healthcare settings has significantly advanced personalized medicine, minimizing adverse effects and enhancing treatment efficacy. AI technologies are able to analyze vast datasets, including genetic information and electronic health care records, to create tailored diagnostic and treatment plans that align with individual patient profiles. AI can identify patterns in complex medical data through machine learning algorithm utilization, facilitating personalized treatment strategies [4]. Techniques such as deep learning enhance diagnostic accuracy, allowing for early disease detection and specific interventions [5]. This could benefit pharmacogenomics, predicting patient responses to medications and outcomes in PEM, leading to trial-and-error reduction. Thus, the integration of AI technologies in PEDs is transforming healthcare delivery by enhancing diagnostic accuracy, treatment customization, and resource management.

Fever is among the most frequent presenting complaints in the PED. While the majority of febrile children are ultimately diagnosed with a self-limiting viral illness, a subset present with serious bacterial infections (SBIs), such as bacterial pneumonia, pyelonephritis, meningitis, or sepsis [6,7]. The management of febrile infants has remained a clinical challenge for more than three decades, as most children with fever have benign viral infections, yet approximately 12% of infants younger than 30 days and 9% of those aged 30–90 days are found to have an SBI [6,8–10]. Infants under 3 months of age are particularly vulnerable due to the immaturity of both innate and adaptive immune responses [11]. The risk of SBI is inversely related to age, reflecting the ongoing development of the innate immune system, which constitutes the first line of defense against pathogens [12]. In this context, AI technologies hold promise for improving diagnostic accuracy by predicting the likelihood of SBI, enabling more personalized clinical decision-making, optimized treatment strategies, and, conversely, reducing unnecessary interventions through refined risk stratification.

In this literature review, we will explore how AI-driven innovations support pediatric emergency care through diagnostic assistance, predictive modeling for febrile disease progression, and patient outcomes. Moreover, we will discuss current challenges and explore future possibilities of AI in personalized PEM.

## Current AI applications in personalized decision-making in pediatric emergency medicine

Children present with symptoms that may vary significantly in presentation and severity compared to adults. Factors such as age, developmental stage, and underlying health conditions influence how children exhibit and respond to illness [13,14]. Therefore, traditional diagnostic algorithms that primarily rely on adult data can lead to misdiagnosis or delayed diagnosis in the pediatric population [15].

### Triage, customized symptom analysis, and diagnostics

AI algorithms in PEM typically involve machine learning and deep learning techniques that analyze historical patient data, clinical presentations, and diagnostic outcomes. By training on pediatric-specific datasets, these algorithms can provide more accurate and age-appropriate diagnoses [15,16]. Machine learning models are trained on vast datasets comprising patient symptoms, lab results, imaging studies, and diagnostics outcomes. Algorithms can learn to identify patterns associated with specific conditions, enabling them to make predictions about the likelihood of various diagnoses based on presented symptoms. A study by Wen and colleagues [17] developed a pediatric bacterial pneumonia model that utilizes chest X-rays (CXRs) and clinical data to classify cases of gram-positive and gram-negative bacterial pneumonia accurately. The algorithm significantly outperformed traditional diagnostic methods, leading to improved diagnostic accuracy and reduced time to treatment. Acute respiratory symptoms are among the most common complaints in PED [18]. Due to significant overlap in the clinical features of viral and bacterial respiratory infections, more accurate predictive models are needed to differentiate between them effectively [19,20]. Most studies primarily focus on analyzing the specificity and/or sensitivity of machine learning predictions for radiological diagnostics or outcomes of acute respiratory disease, such as hospitalization [21]. However, there is a lack of research on symptom-based machine-learning prediction models for specific febrile respiratory conditions in pediatric emergency settings. A study by Patel and colleagues developed a machine learning-based predictive model to assess hospitalization needs for pediatric asthma exacerbation cases utilizing only timely triage data. Achieving an AUC of 0.84, the gradient-boosting machine learning model demonstrated the highest predictive accuracy. Notably, nonclinical data had no impact on model performance. This approach could benefit diverse PED settings globally, particularly in environments with combined pediatric and adult emergency departments or in PEDs lacking observation facilities [22].

Fever is one of the most common symptoms of presentation to PED [23]. In most of the cases, fever is associated with an acute viral infection, which is self-resolving and requires only symptomatic treatment [23,24]. However, some of the conditions need prompt treatment interventions and hospitalization to prevent severe and/or lethal outcomes. Various triage tools are used to identify those who need prompt diagnostics, care, and decisions for medical treatment and/or hospitalization [25,26]. Machine learning-based triage tools have demonstrated significant potential in enhancing the prediction of clinical outcomes for children with acute infections, particularly in low-resource settings. A study by Cacedo-Torres and colleagues developed a machine-learning models which were successful in predicting which PED patients should be admitted to a “Fast Track” for low-complexity patients [27]. To train a model, the study used data from 1205 patients. The results revealed how those models could contribute to reducing waiting times and improving patient care. This development could help divert low-complexity cases away from resource-intensive areas, reducing ED congestion, accelerating care for children with minor illness, and freeing resources for critical ill patients. Kwizera and colleagues [28] developed a random forest model capable of predicting hospital mortality in children using easily obtainable clinical variables, achieving an area under the receiver operating characteristic (ROC) curve of 0.8. The best-performing model used only four variables—age, respiratory rate (RR), capillary refill time, and altered mental state, which achieved the highest predictive accuracy. Nevertheless, it should be tested in larger pediatric populations in both high- and low-middle-income countries. However, low-resource healthcare settings could be the ones that would benefit the most from the application of personalized AI solutions. Hansoti and colleagues [29] introduced the SCREEN (Sick Children Require Emergency Evaluation Now) tool, which effectively identified critically ill children with high sensitivity and moderate specificity, surpassing several conventional triage systems, such as Management of Childhood Illnesses, the Pediatric Early Warning, the Pediatric South African Triage Scale, and the WHO Emergency Triage Treatment Tool. Yehuala and colleagues [30] applied a random forest model in predicting the absence of maternal healthcare-seeking behavior for ARIs in children under 5 in sub-Saharan Africa. After identification of several significant predictors, such as lack of media exposure, living in rural areas, not breastfeeding, poor wealth status, and others, the model showed 88.89% accuracy, a precision of 89.5%, an *F*-measure of 83%, an AUC ROC curve of 95.8%, and a recall of 77.6% in predicting the absence of mother’s healthcare-seeking behavior for ARIs. A study conducted in Ethiopia [31] used an assembled model combining the support vector model (SVM), gradient boosting, and extreme gradient boosting (XGBoost) to predict the determinant of ARIs among children under five. With an accuracy of 86%, it classified ARI cases, including the child’s age, history of diarrhea, and wealth index, among the top predictive factors. In the PED, such integration could support more holistic risk prediction, particularly in communities where social determinants strongly influence outcomes. All these findings highlight the transformative potential of machine learning to optimize pediatric triage and diagnostic accuracy in resource-limited healthcare settings. Moreover, there is a potential for global scalability and adaptability as those models can be trained and refined on local data; they may be adapted to diverse healthcare systems, from high-resource academic hospitals to low-resource rural clinics, thus offering a flexible tool for improving pediatric emergency care. Still, the incorporation of ML-based triage systems into real-time PED decision-making requires seamless integration with electronic health record (EHR) platforms and triage software, which may be lacking in many settings.

SBIs, including sepsis, remain one of the leading causes of mortality among pediatric patients worldwide [32]. Hwang and colleagues, utilizing structured triage data from a nationwide database and using machine learning algorithms, could accurately predict critical illness and hospitalizations among the children visiting PED [33]. They found that the machine learning model was superior to the conventional triage system. This might lead to better patient management and outcomes in PED, resulting in timely interventions for critically ill children [33]. For PEDs, this improvement could translate into earlier recognition of high-risk children, faster escalation of care, and more efficient prioritization of limited resources, thereby reducing preventable adverse outcomes. Naydenova and colleagues emphasized the utility of automated diagnostic algorithms for pneumonia, reporting impressive diagnostic performance with a sensitivity of 96.6% and specificity of 96.4% based on low-cost clinical measurements [34]. The study evaluated a dataset of 1093 children, with 777 diagnosed with pneumonia and 316 healthy controls, based on 47 clinical characteristics. A small set of four key clinical characteristics (temperature, RR, heart rate (HR), and oxygen saturation) were selected. Another model using free text data, vital signs, and demographic data significantly improved the ability to identify patients with suspected infection in the ED, increasing the AUC from 0.67 to 0.86 [35]. The best-performing models used both the chief complaint and the nursing assessment free text along with vital signs, either using a bag-of-words or topic model approach. The researchers preferred the linear support vector machine model over more complex nonlinear models, such as random forests. These findings underscore cost-effective and scalable solutions relying on basic clinical variables, unlocking the potential of routinely collected information on the arrival to the PED and allowing for global applicability.

Febrile children under 3 months of age presenting to PED pose a diagnostic challenge for identifying SBIs. Standard physical examination and routine laboratory tests often lack the specificity and sensitivity needed for accurate SBI prediction [36,37]. Early identification of high-risk patients is critical to initiate timely antibiotic treatment, which can improve outcomes [38–40]. Nevertheless, accurate identification of low-risk infants is equally important to avoid unnecessary interventions and treatments, which may contribute to adverse effects, including prolonged hospitalization or drug-associated complications [41,42]. There are specific tools/criteria to differentiate low and high-risk patients; however, changing epidemiology and novel biomarkers result in decreased adherence to those guidelines. Chiu and colleagues performed a study in three medical centers in Taiwan, including all feverish infants from 0 to 60 days of age. In total, data from 4211 patients were analyzed. The researchers applied three different machine learning algorithms (logistic regression, SVM, and XGBoost) and found performance variation depending on the features used. Among the three models, SVM used the least number of features to achieve the highest AUROC value (0.84 ± 0.03). CRP was the most commonly shared feature among all the models. Nevertheless, all the models (SVM, logistic regression, and XGBoost) outperformed traditional scoring systems (IBI score devised by Aronson and colleagues [43]) and could better stratify low-risk febrile infants [44]. It is essential to mention that they omitted procalcitonin in the modeling as procalcitonin may not be a standard biomarker in some of the PEDs, especially in resourse-restricted settings. Another study by Goto and colleagues [45] included 52,037 ED visits of infants younger than 3 months. Compared to conventional triage approaches, the machine learning methods had higher sensitivity for identifying critical care needs and higher specificity for identifying hospitalization. This study noted that using machine learning in triage could reduce the undertriage of critically ill children and improve resource allocation in PED. However, most studies focus on prediction models using logistic regression, in which variables and/or their combinations could be tested with AI. Chong and colleagues [46] examined a combination of high temperature, tachycardia, and a low Severity Index Score, demonstrating a significant association with SBI in young infants. Given that these clinical parameters are routinely assessed during triage and clinical examination, they could be incorporated into machine learning algorithms for further evaluation. Similarly, Vos-Kerkhof and colleagues [47] highlighted the utility of clinical prediction models integrating clinical signs, urine dipsticks, and laboratory markers to identify SBI in febrile infants. Nevertheless, the researchers recommend it as an adjunct clinical tool only. These studies indicated that embedding ML-driven risk prediction tools into EHR s for real-time decision support during triage and using ML outputs as adjuncts to, rather than replacements for, clinical judgment could provide clinicians with calibrated risk scores to guide discussions and decision-making. Additionally, standardizing data collection should be the priority to maximize model accuracy and interoperability across institutions.

In most cases, children presenting with fever have a self-limiting viral disease that does not need any serious medical intervention and/or thorough follow-up. However, some rare causes of fever should also be considered. Tsai and colleagues, including data from 74,641 febrile children, showed that their model could identify a rare Kawasaki disease (KD) in emergency settings [48]. The XGBoost machine learning method was demonstrated to be accurate and showed 93% sensitivity and 97% specificity to help physicians differentiate children with KD. The model was able to distinguish KD from other febrile illnesses using only objective laboratory tests without needing to rely on subjective symptoms. In a study by Soneji and colleagues, a retrospective review of medical records of patients suspected of MIS-C was performed. Different features were collected. However, the most important ones used by the random forest model to predict MIS-C were procalcitonin, ferritin, proBNP, and CRP [49]. With a sensitivity of 100%, the machine learning model was only 57% specific to identifying patients without MIS-C. However, after the seed and testing set change, the specificity improved to 86%. Nevertheless, there is still a lack of specific studies regarding initial differential diagnostics for the rare causes of fever, as such markers as proBNP or ferritin won’t be routinely performed in feverish children as the COVID-19 pandemic is over. It is essential to highlight that machine learning models can be continuously refined and updated with novel biomarkers or clinical features as medical knowledge and epidemiology evolve. To achieve this, the establishment of robust data-sharing networks between hospitals is crucial in order to generate larger and more representative training datasets for rare febrile conditions. Such collaborative efforts would facilitate prospective validation studies, enabling assessment of how AI-based tools impact real-world diagnostic accuracy, resource utilization, and patient outcomes in rare but clinically significant conditions. However, it can be difficult to train AI models on rare conditions as sufficiently large and balanced datasets can be lacking. This could increase the risk of overfitting and reduce predictive reliability in real-world PED settings.

Machine learning and prognostic modeling are highly dependent on data quality and structure. In the specific context of PEDs, data cleaning and harmonization processes may reveal missing data points or even absent variables. Despite missing data, Lee and colleagues aimed to validate a machine-learning model for predicting SBI among febrile children. External validation using an independent cohort demonstrated the model’s robustness and reliability, achieving an AUROC of 0.950 and an AUPRC of 0.605, revealing its performance in different settings. Furthermore, this model outperformed the conventional logistic regression model in both the derivation and validation cohorts [50]. In a study by Soneji and colleagues [49], the missing data was handled by imputing median values based on the status of the condition they’ve analyzed. Some studies revealed that missing data is linked to healthcare professional practice patterns, especially on the initial triage when the vital sign data can be missing [51,52]. Arnaud and colleagues reviewed 72 studies that applied machine learning in emergency medicine (EM). And revealed that only 21% of them described their missing data strategy [53]. Those studies did not address children with fever in particular. However, they did show the peculiarities of EM settings in general. ED settings, pediatric, adult, or mixed, are always crowded, and situations are highly demanding and stressful; thus, the different sets of data can be missing, especially if the electronic healthcare record system is newly introduced, the staff is new to the system due to different reasons or no clear guidelines being provided for the data handling. There are methods to overcome these shortages (exclusion of specific variables, exclusion of the cases, mean/median imputation, multiple imputation, interpolation, and others); however, if many data points are missing, the machine learning model cannot be adequately trained, especially if the missing data values are critical for the study question. These studies highlight how crucial the implementation of structured and standardized EHR documentation protocols is. This is particularly vital for minimizing variability in data entry and ensuring the consistent capture of critical parameters. This can be achieved through targeted staff training and clear guidelines that emphasize the importance of accurate triage data collection, particularly when new digital systems are introduced.

Numerous SBIs, including sepsis prediction models have been investigated in pediatric intensive care unit (PICU) settings, with potential applicability in PED. However, most machine learning algorithms for sepsis prediction incorporate data points such as specific protein biomarkers or gene expression analyses, which are not routinely conducted in PED [54,55]. Consequently, these models are currently limited in their applicability to emergency settings, where rapid identification of high-risk patients needing urgent intervention, observation, or hospitalization is critical. However, integrating genomic or exome data into these models could enhance future patient care by improving risk stratification and outcome prediction for high-risk individuals in subsequent PED visits. Additionally, such integration could support diagnostic precision, given the rarity of sepsis in febrile, previously healthy children (less than 1% incidence, excluding specific high-risk populations) [56,57]. Papanastassiou and colleagues [58] tested four different machine learning algorithms for classifying patient days as sepsis or nonsepsis based on vital sign data. They encountered an imbalance between nonsepsis and sepsis cases, and the resulting AUC score of 80.2% indicated suboptimal predictive performance, underscoring the need for further model optimization. Liporaci and colleagues [59] developed a machine learning model to detect bloodstream infections in PICU with 99.33% accuracy and 98.89% precision. The sensitivity of this model was 100% sensitive and 98.46% specific. This model included patient demographics, clinical and laboratory data, and time series data on vital signs and lab values. Those changes, even petite, are important and could suggest SBI; however, they are less relevant in PEDs, especially if monitoring in the PICU is more extended. Lamping and colleagues [55] analyzed and validated a diagnostic model using four clinical and four laboratory parameters, differentiating sepsis from noninfectious PICU SIRS. This model outperformed biomarkers such as CRP, IL-6, and PCT; however, it misclassified 28% of noninfectious SIRS cases. Nevertheless, this model, as many previously mentioned, still lacks external validation studies to confirm the generalizability across different populations, healthcare settings, and treatment practices. While most sepsis prediction models have been developed in PICU environments, their adaptation to PED settings holds the potential to transform early recognition of SBIs by leveraging widely available data streams, enabling real-time risk stratification, and providing scalable decision support. Importantly, prioritizing routinely collected clinical and laboratory data over specialized biomarkers may facilitate the development of pragmatic and broadly applicable sepsis prediction tools tailored specifically for pediatric emergency care.

### Pediatric-specific imaging analysis

SBIs include many diagnoses; one of them is pneumonia, which remains a significant concern in pediatric care, with the CXR being among the most performed radiological tests in the PED [60]. Recently, significant advancements have been achieved in medical image analysis tasks using AI and deep learning [61]. Given that widespread availability, CRX images are well-suited for a wide range of computer-based techniques, from simple machine learning to deep convolutional neural networks (CNNs) [62]. Several previous studies have explored the use of AI to identify pneumonia using CXR images and associated clinical data. However, most of these studies concentrated on adult populations even though the presentation of pneumonia or other diseases in children can differ significantly [63]. Additionally, it may vary depending on the causative pathogen and the specific type of pneumonia [64].

Ayan and colleagues demonstrated that AI can be widely applied in medical research and even integrated into daily medical practice [65]. In this field, pneumonia has been a primary focus of a wide range of applications [61]. For example, Lan and colleagues compared multiple CNN models to evaluate *Mycoplasma pneumonia* vs virus-induced pneumonia, analyzing 769 CXR images [66]. The study employed three distinct models for pediatric pneumonia diagnosis: ResNet50, DenseNet121, and EfficientNetv2-S. All models used cross-entropy as the loss function. Data from a single hospital were randomly split into the training, validation, and test sets in a 60:20:20% ratio, while data from the second hospital served as an independent test set. The study found that one of the ResNet50 achieved 80% accuracy on the validation set and an accuracy of 82.65% and 83.27% on the two test sets, with the AUC of 0.822 and 0.758, respectively. The other two models tested showed slightly inferior results. This study highlighted the potential of such AI-driven approaches to be integrated into rapid diagnostics and clinical practice, aiding in the early screening and differentiation of pneumonia in children. The ResNet50 model was also evaluated in a separate study focused on differentiating viral from bacterial pneumonia in pediatric CXRs [67]. Its performance was compared to four human reviewers, achieving comparable diagnostic accuracy using only CXRs. In a systematic review by Field and colleagues, the efficacy of various AI algorithms for pediatric pneumonia diagnosis via CXRs was evaluated across five studies [60]. An ensemble AI algorithm achieved the highest sensitivity (96.3%)—VGG16 and Set C. DenseNet201 demonstrated the highest specificity (94%) and accuracy (95%). The customized VGG16 model attained an AUC = 0.95 for both of the baseline image sets (the original chest radiographs produced and cropped images). Rajaraman and colleagues investigated the performance of the VGG16 model pre-trained on ImageNet [68]. This model provided a strong initialization for learning task-specific features, leading to faster convergence, reduced bias, overfitting, and improved generalization. For classifying bacterial versus viral pneumonia, the customized VGG16 showed similar performance on both baseline and cropped region of interest (ROI) images. However, the cropped ROI yielded better results in the multi-class classification task than the baseline data. In another study, Smith and colleagues developed an algorithm using NLP and a random forest classifier that combined natural language processing and random forest classifiers to identify potential pediatric pneumonia cases from radiology reports [69]. Using approximately 5,000 historical CXR reports, their model achieved an AUC of 0.954, with a sensitivity of 0.899, a specificity of 0.949, and a positive predictive value of 0.781 for detecting pediatric pneumonia. This underscores the effectiveness of AI in real-world clinical care, demonstrating its potential to improve diagnostic performance and streamline healthcare workflow. Furthermore, AI systems can serve as a reliable “second reader,” which is particularly valuable during periods of high patient volume or in settings with limited access to pediatric radiologists. In addition, integrating imaging, laboratory, and clinical data could further strengthen risk stratification and support more informed disposition decisions, such as admission versus safe discharge. Of note, there could be some limitations for implementation due to variable image quality or annotation bias, as training labels often rely on clinician interpretation, which itself may be inconsistent. In addition, initial setup, maintenance, and ongoing updates for AI platforms may be financially prohibitive in certain healthcare systems. Moreover, training models solely to distinguish viral from bacterial pneumonia can be challenging, primarily because of the risk of co-infection, which has been reported in up to 20%–30% of pediatric pneumonia cases [70,71]. Additionally, several other conditions—including bronchiolitis, atelectasis, pulmonary edema, and even noninfectious inflammatory diseases—can produce CXR findings that mimic pneumonia, thereby reducing model specificity [72]. These factors highlight the complexity of developing reliable AI-based diagnostic tools that rely exclusively on CXR interpretation..

With increasing recognition of ultrasound (US) as a valuable tool for accurate, noninvasive diagnostics in the PED, point-of-care ultrasound (POCUS) has become a reliable method for evaluating respiratory complaints, often outperforming conventional CXRs in diagnosing pneumonia [73]. Specific abnormalities such as sparse B-lines, confluent B-lines, pleural abnormalities (e.g., pleural effusion), and large consolidations (>1–1.5 cm) were found to be more prevalent in bacterial infections, while children with viral and atypical pneumonia had significantly more vertical deep artifacts (B-lines) [74,75]. Large consolidations and pleural effusion were exclusively observed in bacterial infections. Amatya and colleagues performed lung US and evaluated the accuracy of diagnosing pneumonia in children [76]. Lung US had a sensitivity of 89.3% (95% CI 81–95), specificity of 86.1% (95% CI 82–90), positive likelihood ratio of 6.4, and negative likelihood ratio of 0.12 in predicting pneumonia. However, POCUS remains highly user-dependent, posing challenges for less experienced physicians. A study [77] conducted in the USA and published in 2022 explored the use of an AI-enhanced pleural sweep to generate panoramic views of the lungs, assisting novice clinicians in diagnosing pneumonia. The study reported a sensitivity of 66.7% (95% CI: 9.4%–99.1%), a specificity of 96.5% (CI: 82.2%–99.9%), and an overall diagnostic accuracy of 93.7% (CI: 79.1%–99.2%). The average image quality rating across lung fields was 2.94 (±0.16) out of 5, and interrater reliability among expert sonographers was strong, with a kappa coefficient of 0.8. These findings, along with results from other studies, highlight the potential of AI-assisted tools to enhance lung US diagnostics [78,79]. By reducing operator dependency, standardizing diagnostic accuracy, and enabling broader implementation of POCUS, AI can help PEDs improve the diagnosis and management of pediatric pneumonia, ultimately enhancing patient outcomes and resource efficiency.

As AI technology evolves, it can simplify complex tasks, enabling young physicians and novice sonographers to make rapid and accurate medical decisions, particularly in resource-limited settings. Correa and colleagues [80] demonstrated the feasibility of training an artificial neural network to detect pneumonia infiltrates in lung US. Despite the study’s limited sample size, their model achieved a sensitivity of 90.9% and a specificity of 100% in identifying pneumonia-related consolidations. This underscores the potential for AI algorithms to automate the detection of lung pathologies. Additionally, AI has been shown to guide novice users in acquiring high-quality bedside cardiac US images, further supporting its utility in training and diagnostics [81]. These advances are particularly valuable in low-resource environments, where expert interpretation of US images is often unavailable. Mobile bedside US (mBSUS) is a potentially valuable diagnostic tool integrated with AI automated interpretation. Camelo and colleagues assessed its diagnostic accuracy by comparing CXR and mBSUS in children aged 1–59 months diagnosed with severe/very severe pneumonia [82]. Among the 11 patients enrolled in the study, mBSUS identified pulmonary abnormalities in three cases that were either undetected or barely visible on CXRs. A machine learning-based platform can aid in clinical decision-making by integrating image recognition, clinical signs, and laboratory test results to provide scientific and efficient diagnostic and treatment methods [83]. This could be particularly valuable in settings where access to expert radiologists or sonographers is limited. AI can support both image acquisition and interpretation, enabling broader use of POCUS among clinicians with varying levels of expertise. Such integration may also shorten hospital stays by reducing reliance on advanced imaging and facilitating rapid bedside diagnostics. However, not all PEDs have access to portable US devices, and despite AI assistance, image quality still depends heavily on the operator’s technique.

AI is increasingly applied in pediatric musculoskeletal medicine, offering significant potential in diagnosis, treatment, and management. POCUS is a particularly valuable tool for diagnosing other causes of SBI, and it is likely to benefit from AI integration in the near future [84].

One of the infections, osteomyelitis, often presents with nonspecific signs and symptoms, requiring a high degree of clinical suspicion for accurate differential diagnosis. This typically requires a combination of diagnostic tests, including laboratory tests, X-rays, and MRI. However, these may not always be immediately available [85]. Tsung and colleagues presented a case series where POCUS was used as a first-line diagnostic tool in the PED for children with suspected osteomyelitis [86]. This underscores the importance of employing POCUS early in evaluating suspected pediatric osteomyelitis, particularly in resource-limited settings where advanced imaging may be inaccessible, to enable timely treatment and reduce complications. Key sonographic findings for osteomyelitis include cortical disruption, periosteal elevation, and abscess formation, with these findings varying based on disease duration and severity. Notably, POCUS can detect osteomyelitis several days before radiographic findings, allowing for earlier diagnosis and intervention [87]. Leg pain and limping are also common reasons for pediatric emergency visits, with toxic synovitis being one potential cause [88]. Diagnosing toxic synovitis often requires multiple tests, which can prolong hospital stays [89]. A study comparing two groups of limping children—those who underwent POCUS versus those who did not—found that the POCUS group experienced an average reduction in hospital stay by nearly an hour [90]. Thus, incorporating POCUS into the early evaluation of musculoskeletal complaints in the PED could enhance the detection of SBIs, reduce unnecessary imaging and hospitalizations, and improve overall efficiency in patient management. However, its implementation is limited by variability in interpretation and the absence of standardized guidelines, which may reduce reproducibility across institutions. In addition, POCUS may not always be feasible in high-volume emergency settings.

However, while AI offers promising potential, it also introduces new challenges. A significant issue is that imaging data and its reports are often unstructured, making preparing such data for AI analysis difficult. This process is not only inefficient but also susceptible to bias [15]. Neural networks and deep learning models have been explored to automate the interpretation of unstructured imaging data [91], however, pediatric data adds additional complexity. Variations in age, physiology, disease risks, and age-related pathophysiological changes must be accounted for to ensure accurate AI-based models, further complicating automated processing [92]. Another major concern is data privacy. To address this, some studies advocate for using federated learning methods, which allow AI models to learn from decentralized data without requiring direct data sharing. This approach minimizes the risk of privacy leakage compared to traditional machine learning methods, offering a more secure solution for leveraging AI in healthcare [93].

Although AI demonstrates promising potential in medical diagnostics, challenges such as unstructured data, variability in pediatric cases, and privacy concerns highlight the need for further development, broader application, and robust datasets to ensure accuracy, efficiency, and reliability in clinical practice.

## Conclusion

AI models using pediatric-specific datasets can enhance diagnostics precision by analyzing clinical symptoms, lab results, and imaging, enabling timely and accurate interventions for febrile illnesses. Those tools demonstrated the potential to effectively identify critically ill children, stratify risks, and improve resource allocation, particularly in low-resourse settings, reducing overcrowding and enhancing patient care. Some deep learning models, like ResNet50 and VGG16, improve pediatric pneumonia diagnosis, enabling early screening and disease differentiation. Integration of AI with POCUS was observed to boost diagnostic accuracy and support novice clinicians. However, AI adoption in PEDs is hindered by missing data, reliance on nonroutine biomarkers, and limited model validation, underscoring the need for standardized data practices and robust modeling techniques. Additionally, unsolved privacy concerns persist, prompting the exploration of methods for secure AI deployment.
